# Rates of colorectal cancer detection in screening colonoscopy post appendicectomy in patients 50 years and over

**DOI:** 10.1016/j.amsu.2018.11.012

**Published:** 2018-11-20

**Authors:** Sunny Dhadlie, Daniel Mehanna

**Affiliations:** Caboolture Hospital, 120 McKean Street, 4510, Queensland, Australia

**Keywords:** Colorectal cancer, Appendicitis, Appendices cancer, Colonoscopy

## Abstract

**Introduction:**

Acute appendicitis in older adults is relatively uncommon and could be the first presentation of an underlying colorectal carcinoma. Colonoscopy in these individuals affords the opportunity for earlier diagnosis and treatment. The finding of increased rates of colorectal cancer (CRC) with older patients who have had appendicitis was supported by a number of small studies and case reports in the early 1980s.

With the advent of CT scanning and laparoscopic appendicectomy, increased ability to visualize the caecum has been achieved.

**Purpose:**

A retrospective 12-month study of all patients presenting with acute appendicitis aged 50 years and over from 1st May 2017 to 31st May 2018, and review of post operative screening colonoscopy findings.

**Results:**

Forty-three patients met inclusion criteria. The patients’ median age was 62 years (range 50–85 years). 47% of the patients were male. 86% of patients had abdominal CT scans prior to surgery with acute appendicitis visualized in 97% of these cases. Acute appendicitis was found in 100% of cases with no clinical suggestion of CRC operatively or pathologically. 46% of patients had pertinent findings on colonoscopy. This included a malignant obstructing tumour at the hepatic flexure and a tubular adenoma in the transverse colon in a second patient. The remaining findings in this cohort of patients included diverticular disease and benign polyps.

**Conclusion:**

Despite the advancement in visualization of anatomy with CT scan and laparoscopic appendicectomy there is still a role for screening colonoscopy in patients greater than 50 years of age with appendicitis particularly if they have associated bowel symptoms or risk factors for CRC.

## Highlights

• Acute appendicitis in older adults is uncommon, may represent first presentation of an underlying malignancy.
• CT scans and laparoscopic surgery enable enhanced visualisation of the appendix compared with previous open surgical technique.
• Appendiceal adenocarcinoma is rare with an incidence of 0.08 - 0.2%. Synchronous colonic malignancy is found in up to 3% of patients with appendiceal tumours.

## 1 Introduction

Acute appendicitis in older adults is relatively uncommon. The presentation of acute appendicitis in these individuals may be the first presentation of an underlying malignancy. Screening in this cohort of patients affords the opportunity for earlier diagnosis and treatment.

With the advent of CT scans and laparoscopic surgery for the management of acute appendicitis the utility of screening individuals post operatively has been questioned [1].

There appears to be significant dichotomy amongst general surgeons in regards to colonic investigation following appendicitis in older individuals [2].

This case has been reported in line with the SCARE criteria [3].

## 2 Materials and methods

A retrospective 12-month study of all patients presenting with acute appendicitis aged 50 years and over from 1st May 2017 to 31st May 2018, and review of post operative screening colonoscopy findings in this cohort.

Forty-three patients met inclusion criteria. The patients’ median age was 62 years (range 50–85 years). 47% of the patients were male.

This case has been reported in line with the PROCESS criteria [3].

researchregistry4408.

## 3 Theory

Shears first postulated the relationship of right-sided colon cancer presenting with acute appendicitis in 1906 [4]. This has been further supported by the work of Lai HW et al. in demonstrating the increased risk of underlying CRC in older adults with appendicitis [5]. Colonoscopy in patients whose histology from appendicectomy was benign is controversial.

## 4 Results

86% of patients had abdominal CT scans prior to surgery with appearances suggestive of acute appendicitis in 97% of cases. Acute appendicitis was found intra-operatively and histologically. 23% of operative cases revealed a perforated appendix. There was no evidence of dysplasia or malignancy on histology. 46% of patients had findings on post-operative screening colonoscopy of these only 1% had findings concerning for malignancy.

There was a malignant obstructing tumour at the hepatic flexure in one patient. This patient had previously had an abdominoperineal resection (APR) for rectal cancer and duodenal MALT B cell lymphoma. The case was confounded given the patient has already been treated for rectal cancer previously, therefore having greater risk for recurrence of malignancy.

The second patient had a tubular adenoma with high-grade dysplasia in the transverse colon. The remaining findings in this cohort of patients included diverticular disease and benign polyps.

2.3% of patients were smokers, and 11.6% had a personal history of cancer.

4.6% of patients had a history of previous CRC that was treated successfully (both cases were rectal cancer, one treated with an APR and the other with an ultra-low anterior resection). The second case identified dysplastic changes in a polyp in the transverse colon, which arguably without screening colonoscopy could have developed into malignant mass that may only have been detected when the patient became symptomatic.

The remaining patients who had colonoscopy did not have clinical, operative or histological findings concerning for malignancy.

## 5 Discussion

Given the improvement in the modality of CT scanning it has been increasingly relied upon to avoid unnecessary invasive investigations.

In this study of all the patients who had pre-operative CT scans none were concerning for malignancy, despite one patient having an obstructive tumour at the hepatic flexure detected on colonoscopy.

CT scan is actually a poor diagnostic tool for detection of colonic malignancies with only 70% sensitivity with an unprepared bowel and even less in the setting of acute appendicitis [6,7].

Appendiceal adenocarcinoma is rare with an incidence of 0.08–0.2%. Synchronous colonic malignancy are found in up to 3% of patients with appendiceal tumours – these circumstances warrant complete colonic investigation for planning of definitive surgery [8,9].

CRC may cause acute appendicitis either by way of direct obstruction of the appendiceal lumen or as result of oedema and inflammation. In addition partial downstream colonic obstruction may result in an increase in luminal pressures, therefore predisposing to acute appendicitis. An alternate pathogenesis is that of immune-mediated lymphoid hyperplasia associated with malignancy leading to appendiceal obstruction and appendicitis [10,11].

It has been postulated with much debate that appendicectomy may also be a contributing factor to CRC in some patients given its immune function.

The relationship between acute appendicitis in patients greater than 40 years of age and readmission for CRC was studied Arnbjornsonn et al. and found to be 2.9% in 3 years [12].

The odds ratio of CRC in patients greater than 40 years of age with acute appendicitis has been reported to be as high at 38.5 fold.

Current bowel screening guidelines recommend colonoscopy for those 50 years and over with bowel symptoms or risk factors.

This study has shown that detection of appendicitis in those greater than 50 years of age is not an independent predictor of colorectal cancer. In those who had colorectal cancer a history of previous malignancy was noted. Therefore, there is no evidence to screen this age group with colonoscopy post appendicectomy unless the patient exhibits bowel symptoms or risk factors for CRC. Patients who have histology supporting appendicitis and no additional risk factors could be offered faecal occult blood testing (FOBT) to assess whether they would warrant a colonoscopy for further investigation.

Prospective studies should be conducted to analyse appendicitis in those greater than 50 years of age and the link between CRC detection on colonoscopy with healthy patients with no other risk factors for malignancy so a tangible link between appendicitis and colorectal cancer can be made. Studies with a longer follow up time post appendicectomy would be valuable to assess if there is a delayed incidence of CRC. More discerning selection of patients for colonoscopy would in addition decrease the risk of potential adverse events related to the procedure and reduce health care costs.

## 6 Conclusions

Despite the known increased risk of underlying CRC in patients 50 years and over who present with acute appendicitis, there are few cases detected in routine colonoscopic investigation of all patients.

Colonoscopy should be offered to those with appendicitis aged greater than 50 years who have bowel symptoms or are particularly high risk, such as those with previous malignancy.

There are no conflicts of interest or sources of funding.

## Provenance and peer review

Not commissioned, externally peer reviewed.

## Ethical approval

This study is exempt from ethical approval in this institution.

## Sources of funding

There are no sources of funding for this research.

## Author contribution

Dr Sunny Dhadlie.

Study concept.

Data collection, analysis, interpretation.

Writing the paper.

Contributers:

Dr Daniel Mehanna.

Study concept.

## Conflicts of interest

There are no conflicts of interest including employment, consultancies, stock ownership, honoraria, paid expert testimony, patent applications/registrations, grants or other funding.

## Research registration number

researchregistry4408.

## Guarantor

Dr Daniel Mehanna.

## Consent

Written informed consent was obtained from the patients for publication of this case report and accompanying images. A copy of the written consent is available for review by the Editor-in-Chief of this journal on request.

## Appendix A Supplementary data

Process Checklist Process Checklist
